# Antigen Presentation in the Lung

**DOI:** 10.3389/fimmu.2022.860915

**Published:** 2022-05-09

**Authors:** Takumi Kawasaki, Moe Ikegawa, Taro Kawai

**Affiliations:** Laboratory of Molecular Immunobiology, Division of Biological Science, Graduate School of Science and Technology, Nara Institute of Science and Technology (NAIST), Ikoma, Japan

**Keywords:** lung, antigen presentation, antigen cross presentation, dendritic cells, macrophages

## Abstract

The lungs are constantly exposed to environmental and infectious agents such as dust, viruses, fungi, and bacteria that invade the lungs upon breathing. The lungs are equipped with an immune defense mechanism that involves a wide variety of immunological cells to eliminate these agents. Various types of dendritic cells (DCs) and macrophages (MACs) function as professional antigen-presenting cells (APCs) that engulf pathogens through endocytosis or phagocytosis and degrade proteins derived from them into peptide fragments. During this process, DCs and MACs present the peptides on their major histocompatibility complex class I (MHC-I) or MHC-II protein complex to naïve CD8^+^ or CD4^+^ T cells, respectively. In addition to these cells, recent evidence supports that antigen-specific effector and memory T cells are activated by other lung cells such as endothelial cells, epithelial cells, and monocytes through antigen presentation. In this review, we summarize the molecular mechanisms of antigen presentation by APCs in the lungs and their contribution to immune response.

## Introduction

The lung is the peripheral tissue that exchanges gas during respiration; therefore, it is exposed to the outer environment, which potentially increases the risk of invasion by viral and bacterial pathogens. Respiratory viruses, including influenza virus and recent coronavirus, induce inflammation and tissue damage, leading to disorders of the lungs. The high infectivity and spreadability of these viruses have caused a worldwide pandemic in recent years and has provoked the argument for recurrent infection and efficacy of vaccination in order to suppress the pandemic. Innate immune cells such as dendritic cells (DCs) and macrophages (MACs) in the lungs form the first line of defense by recognizing the molecular structures common to pathogens, called pathogen-associated molecular patterns, through pattern recognition receptors (1, 2). During the past decade, various types of lung DCs and MACs have been identified and classified according to surface markers, expression genes, and corresponding transcription factors with specialized functions. These DCs and MACs function as antigen-presenting cells (APCs) that engulf pathogens through endocytosis or phagocytosis and present their peptides on major histocompatibility complex class I (MHC-I) or MHC-II protein complex to naïve CD8^+^ or CD4^+^ T cells, respectively. Although DCs and MACs are known as professional APCs with a higher expression of co-stimulatory molecules, such as CD80 and CD86, other types of cells such as monocytes and epithelial cells in the lungs also have the potential to present antigens to T cells.

**Figure 1:**
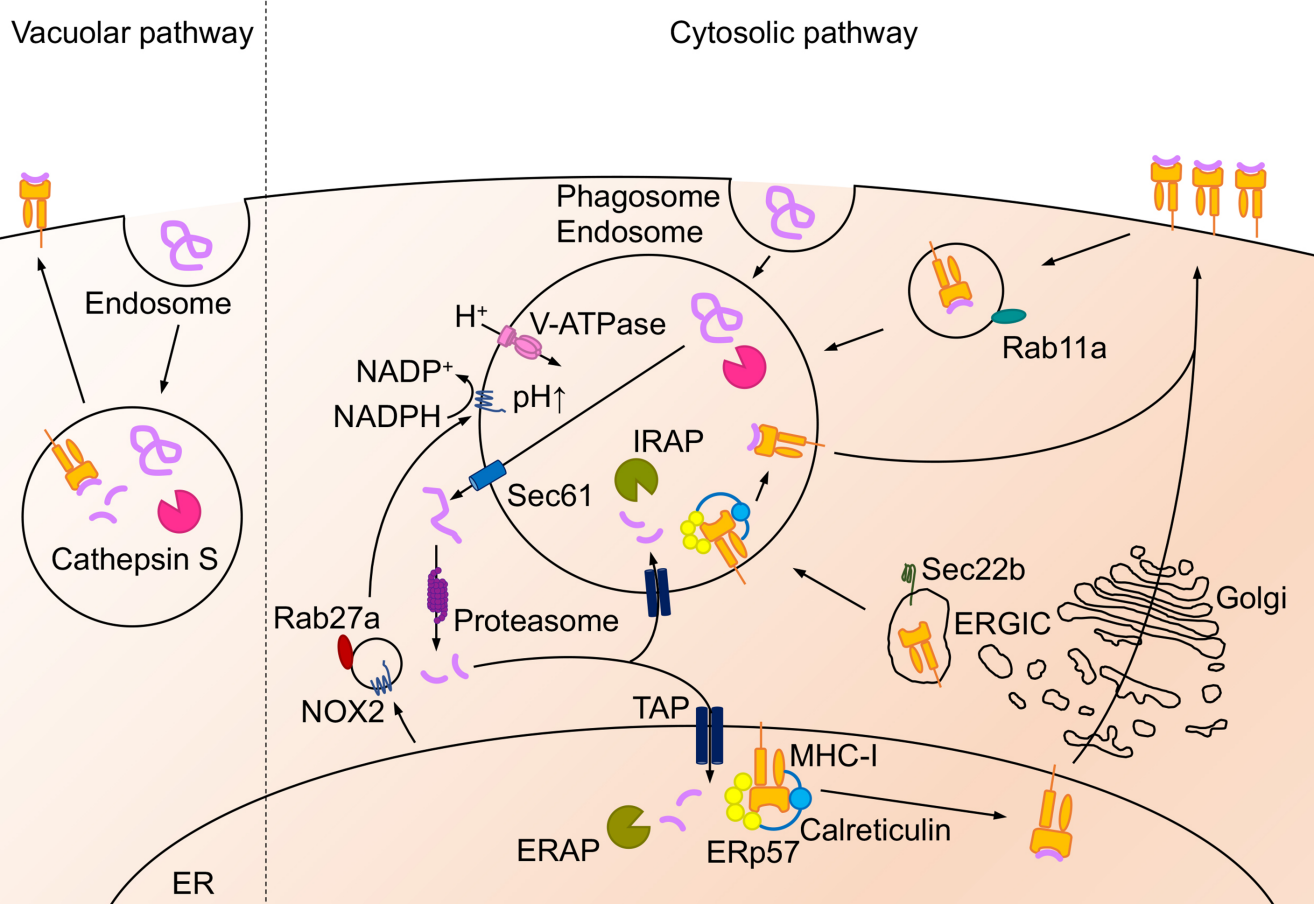
Antigen presentation on MHC-II molecule. Extracellular antigens are endocytosed or phagocytosed, and intracellular antigens are translocated to the late-endosome or the lysosome *via* autophagosome- or LAMP-2A- mediated autophagy. Then these antigens are degraded by asparaginyl endopeptidase and cathepsin. MHC-II is synthesized in ER and mainly pooled at the plasma membrane as MHC-II-Ii chain complex. When the complex translocates from the ER or the plasma membrane to the acidic compartment, Ii chain is degraded into CLIP and driven out by interaction with H2-M. Afterward, antigen peptides bind to the MHC-II and the peptide-MHC-II complex exports to the cell surface.

**Figure 2:**
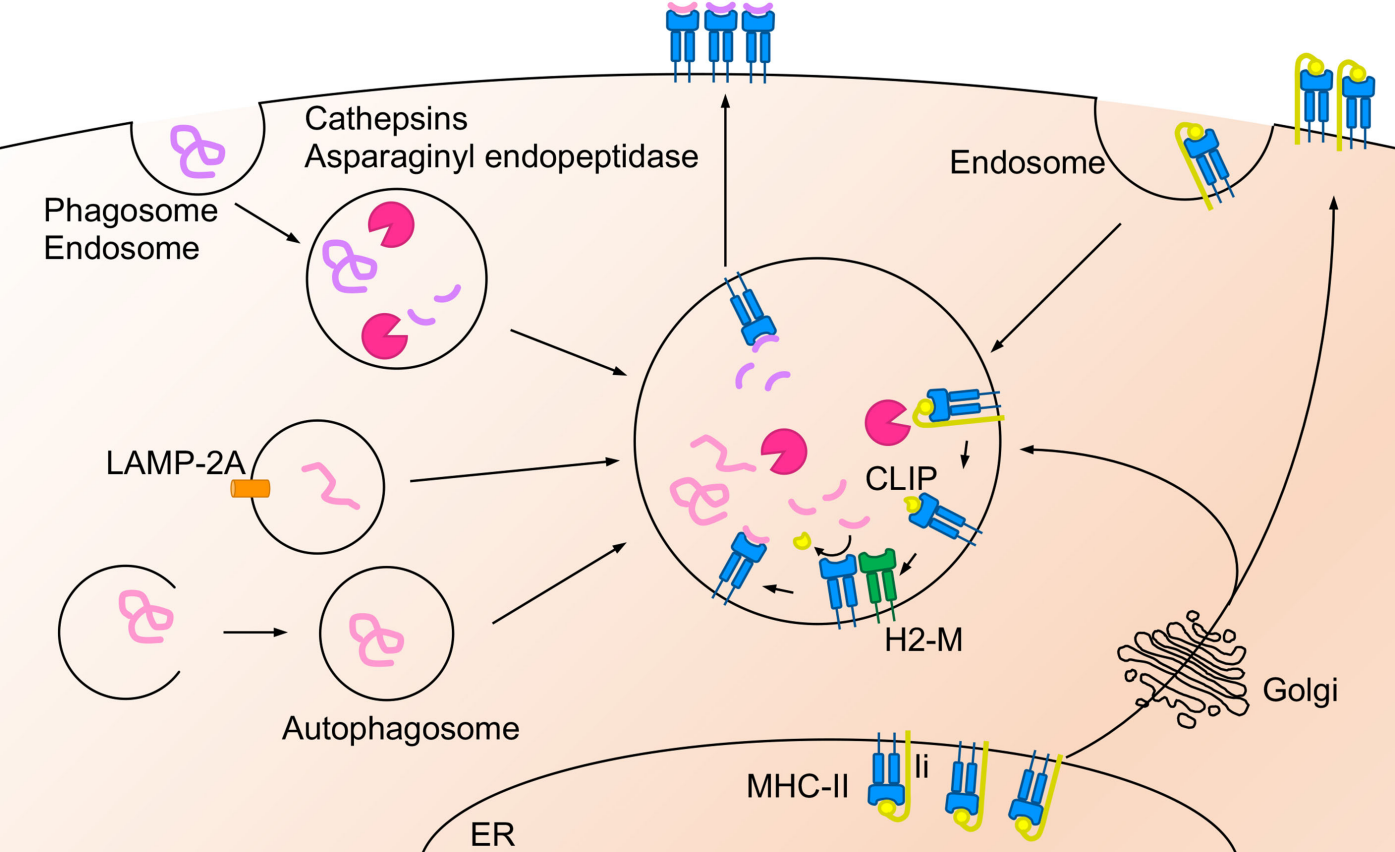
Antigen cross-presentation on MHC-I molecule. Extracellular antigens are presented *via* “vacuolar pathway” or “cytosolic pathway” in the cross-presentation pathway. In the vacuolar pathway, endocytosed antigen peptides are degraded by cathepsin S and bind to MHC-I in the endosomal compartment. In the cytosolic pathway, endocytosed or phagocytosed extracellular antigens are translocated to the cytosol *via* Sec61 and degraded by proteasome. The degraded peptides are transported into the ER or the endosome *via* TAP and trimmed by ERAP (in the ER) or IRAP (in the endosomes). TAP form PLC with MHC-I, ERp57 and calreticulin. Afterward, the trimmed peptides bind to the MHC-I and transported to the cell surface. The MHC-I in the endosomes is recruited from the plasma membrane through Rab11a^+^ recycle endosome, the ER, or the ERGIC. Antigen degradation regulated by the acidification in the endosome, the phagosome, and the lysosome by V-ATPase. On the other hand, NADPH oxidase NOX2 regulates phagosomal alkalization and is recruited to the phagosomes by Rab27a-dependent pathway.

APCs load peptides derived from exogenous antigens on MHC-II and present peptide-MHC-II complex to CD4^+^ T cells whereas APCs load peptides derived from both endogenous and cytosolic antigens on MHC-I and present peptide-MHC-I complex to CD8^+^ T cells (**Figures 1**, **2**). In addition, specific APCs take up exogenous antigens, process them, and load peptides onto MHC-I to CD8^+^ T cells, a process called antigen cross-presentation (3). Lung DCs are largely divided into three major subsets: cDC1s, cDC2s, and plasmacytoid DCs (pDCs). These DCs have been focused on as key regulators of T cell responses (4); however, recent evidence indicates that other types of cells in the lung, such as MACs, monocytes, and epithelial cells, also have antigen presentation capacity to both CD4^+^ and CD8^+^ T cells. MACs in the lung are mainly classified into alveolar macrophages (AMs) and interstitial macrophages (IMs). Lung epithelial cells (LECs) consist of alveolar type I (ATI) and alveolar type II (ATII) cells in the alveoli, and the predominant cell types constituting the bronchial airway epithelium include endothelial cells, basal progenitor cells, ciliated cells, secretory club cells, and goblet cells (5, 6). Lung DCs, MAC and LECs express MHC-I and/or MHC-II on their cell surface and potentially present antigen to CD4^+^ or CD8^+^ T cells (7).

During pathogen infection in the lung, pathogen-specific CD4^+^ and CD8^+^ cells are primed in the lung-draining lymph nodes by antigen-presenting DCs that migrate from the infected area in the lung (8, 9). Antigen-presenting DCs encounter naïve CD4^+^ and CD8^+^ T cells in the lymph nodes, where antigen-specific T cells are selected, and the proliferation and differentiation by antigen presentation on MHC molecules are induced along with the assistance of co-stimulatory molecules and the local cytokine environment (10, 11). Antigen-specific CD4^+^ and CD8^+^ T cells in the lymph nodes migrate to the lungs to directly eliminate infected cells or induce the accumulation of other immunological cells for pathogen clearance. In addition, antigen-specific T cells encounter local APCs in the lungs, including DCs, MACs, monocytes, and LECs, and further differentiate and expand in the lung (12). Parts of antigen-specific cells differentiate into long-lived memory cells, which are divided into three types of population: central memory T (T_CM_) cells, which are largely found in secondary lymphoid organs; effector memory T (T_EF_) cells, which systematically circulate, transiently entering peripheral tissue, and resident memory T (T_RM_) cells, a non-circulating, self-renewing population located in peripheral tissues including the lungs (13, 14). There has been increasing evidence that antigen-specific memory T cell formation through antigen presentation or cytokines is facilitated by various types of lung cells. In this review, we summarize the molecular mechanisms of antigen presentation to MHC-I and MHC-II on APCs and memory T cell formation by APCs during pathogen infection in the lung.

### Molecular Basis of Antigen Presentation to CD4^+^ T Cells

In general, extracellular antigens are endocytosed or phagocytosed by APCs and degraded by proteases such as asparaginyl endopeptidase (15) and cathepsins S, B, H, and L (16–18). Degraded peptides are ultimately presented on MHC-II molecules to prime CD4^+^ T cells (19) (**Figure 1**). However, less than 30% of antigens on MHC-II are derived from endogenous antigens, such as cytoplasmic or nuclear antigens (20, 21). Regardless of peptides derived from self or non-self-antigens, these peptides can be presented by APCs, non-professional APCs, or tumor cells mainly *via* autophagosome- or chaperone-mediated autophagy (22). Antigen degradation is mediated by the fusion of autophagosomes with endosomes and lysosomes in autophagosome-mediated autophagy (23). Antigens degraded by the proteasome in the cytosol are translocated to the late endosome or lysosome, which is enhanced by lysosome-associated membrane protein 2A (LAMP-2A) (24).

Newly synthesized MHC-II forms a complex with the invariant (Ii) chain in the endoplasmic reticulum (ER), and is pooled in the ER or plasma membrane and then respectively, translocated to the endosomes and lysosomes either directly (25) or indirectly though endocytosis (26, 27); however, the complex cannot bind to antigen peptides (28, 29). The Ii chain is degraded into a small fragment called class II-associated Ii chain peptide (CLIP) and binds to MHC-II in the late-endosome or the lysosome (30). The CLIP on MHC-II is driven out by interaction with another nonconventional MHC-II, called HLA-DM in humans and H2-M in mice (30). Then, MHC-II complexes can bind to antigen peptides and be presented on the cell surface (30). The expression of the peptide-MHC-II complex on the cell surface and its turnover by ubiquitination in DCs is essential for their ability to efficiently prime CD4^+^ T cells (31, 32).

### Molecular Basis of Antigen Cross-Presentation Pathway

Specific APCs are thought to take up extracellular antigens through endocytosis or phagocytosis and load peptides onto MHC-I for presentation to CD8^+^ T cells, a process called antigen cross-presentation (3). The extracellular antigen degradation pathway is mainly divided into the “vacuolar pathway”, through which the peptide is degraded in the endosome, and the “cytosolic pathway” which is responsible for the transport of degraded protein through SEC61 from the endosome to the cytosol (33) (**Figure 2**).

#### Vacuolar Pathway of Antigen Cross-Presentation

In the vacuolar pathway, extracellular antigens are endocytosed by APCs and degraded into peptide fragments by proteases in the compartment. Cathepsin S plays a crucial role in antigen degradation in the endosomes of bone marrow-derived DCs (BMDCs) (34). It has been shown that cathepsin S plays a key role in priming CD8^+^ T cells to Influenza A virus (IAV) peptides loaded on MHC-I in the vacuolar pathway (34). In DCs, cathepsin S is also a crucial protease for MHC-II-dependent presentation to CD4^+^ T cells (18, 35) whereas cathepsin L in the thymic cortical epithelium (35) and cathepsin F in macrophages likely correspond to proteases in the vacuolar pathway (36). The degraded peptide by cathepsins forms a complex with MHC-I in the endosome, and the peptide-MHC-I complex is transported to the cell surface. However, it is not clear whether cathepsins are required for antigen degradation in all lung APCs during pathogen infection.

#### Cytosolic Pathway of Antigen Cross-Presentation

In the cytosolic pathway, phagocytosed or endocytosed antigens are translocated from the endosomal compartments to the cytosol *via* Sec61 (33) and degraded to peptide fragments by the proteasome in the cytosol (37, 38). Phagosomes and endosomes are mainly acidified *via* V-ATPase for degradation (39), which is regulated by Toll-like receptor (TLR) signals and other maturation signals (40), and restriction of antigen in these compartments by acidification is important for peptide degradation in the cytosolic pathway. DCs lacking the NADPH oxidase NOX2 show enhanced phagosomal acidification and increased antigen degradation, resulting in impaired antigen presentation (41, 42). The recruitment of NOX2 to these compartments is prevented by deficiency of Rab27a, which causes acidification of phagosomes, limiting antigen degradation (43).

The cytosolic pathway is further categorized to two pathways; “ER-dependent pathway” and “Endosomal pathway”. The ER-dependent pathway is the most common route to ER for antigen peptide. Antigen peptides in the cytosol are transported into the ER mainly through transporter associated with antigen processing (TAP) and form peptide-MHC-I complexes in the ER. On the other hand, peptides degraded by the proteasome in the cytosol are transported back to the endosomes through TAP in the endosomal pathway. MHC-I molecules are recycled in the cells. MHC-I molecules in the endosome are transported from the plasma membrane through the Rab11^+^ recycling endosomes (44) and are also recruited from the ER or the ER-Golgi intermediate compartment (ERGIC) (3, 45). Transported peptides are loaded on MHC-I by the peptide loading complex (PLC) the in the ER or the endosomes (46). PLC consists of TAP, oxidoreductase ERp57, MHC-I heterodimer, and calreticulin (46). PLC is recruited to phagosomes or endosomes *via* the Sec22b-ERGIC pathway (47). PLC is also recruited from the recycle endosomes after TLR activation (44). In contrast, the N terminal anchor residues of the peptides are trimmed by ER-resident N-aminopeptidases (ERAP1 and ERAP2 in humans, and ERAAP in mice). Insulin regulated aminopeptidase (IRAP), an aminopeptidase similar to ERAP, trims the peptide in the endosomes (48, 49). These peptide trimming proteins are crucial for efficient antigen peptide binding to MHC-I and contribute to cross-presentation (50–52). Although cytosolic peptides shuttle into the ER through TAP1 in the cytosolic pathway, TAP1 blockade in DCs leads to antigen presentation by MHC-I translocation from ERGIC in a Sec22b-dependent manner rather than the Rab11^+^ recycle-endosome pathway (53).

### DCs and MACs in the Lung

DCs in the lung consist of heterogeneous subsets that exert different functions (54, 55). Lung DCs are largely divided into three major subsets and are broadly subdivided into plasmacytoid DCs (pDCs) and conventional DCs (cDCs). Murine cDCs express high levels of integrin CD11c and are further divided into CD103^+^ DC and CD11b^+^ DCs. CD103^+^ DCs and CD11b^+^ DCs are also referred to as cDC1s and cDC2s, respectively (55–58). Although CD11b and CD11c have been utilized for the separation of DC population, cDCs separation was proposed as two main subsets cDC1s and cDC2s based on the transcription factor expression (59, 60). Interferon regulatory factor 8 (IRF8) and Batf3 drive the development of cDC1s which are separated as XCR1^+^Cadm1^+^CD172a^−^ cDC1s (61–69). On the other hand, IRF4 drives the development of cDC2 which are separated as XCR1^−^Cadm1^−^CD172a^+^ cDC1s (67, 69–76). pDCs develop in the presence of transcription factor 4 (E2-2) and the Ets family transcription factor Spi-B (77–79). In the steady-state, cDC1s associate with airway rather than alveoli in the lung (80, 81). cDC2s are located in the airway and lung parenchyma (82–84). Monocyte-derived DCs (moDCs) have been described as another DC population that accumulates in the lungs during inflammation and viral infection (85–87). MoDCs are also known as inflammatory DCs and monocyte derived cells (88–91). These DCs are subdivided based on the presence of surface markers and recent progress in the technology for single-cell RNA sequencing revealed that the cDC2s population in the lung is subdivided based on expression markers with functional differences, whereas pDCs and cDC1s are a unique population (92–94).

MACs in the lungs consist of two major populations: alveolar MACs (AMs) and interstitial MACs (IMs). AMs are located in the alveolar space of the lungs and are in close contact with the type I and II epithelial cells of the alveoli. AMs are the first line of defense against pathogens for host defense in the lung, with a higher engulfment capacity against antigens and pathogens (95). AMs produce cytokines such as TGFβ, IL6, and type I interferon during pathogen infection and inflammation (95, 96). In addition, AMs play a central role in homeostasis and tissue remodeling. Pulmonary surfactant is a mixture of lipids and proteins secreted into the alveolar space by AT II cells. The surfactant is covered with an interface of alveolar epithelial cells in the lungs to reduce the physical tension during breathing. In addition, the engulfment of surfactant and cell debris by AMs is important for the clearance and maintenance of lung homeostasis. Accumulation of pulmonary surfactant in the absence of AMs causes the development of pulmonary alveolar proteinosis (PAP) (97–99). GM-CSF and TGF-β induce PPAR-γ, a crucial transcription factor for AM development (100). Interstitial macrophages (IMs) reside in the parenchyma between the microvascular endothelium and alveolar epithelium. However, compared with AMs, the role of IMs in lung homeostasis remains poorly understood. Like AMs, IMs engulf bacteria and foreign particles and secrete IL-1, IL-6, IL-10, and TNFα (101–104). IMs form a heterogeneous population that is further subdivided based on surface markers with distinct functions (103, 105).

**Figure 3:**
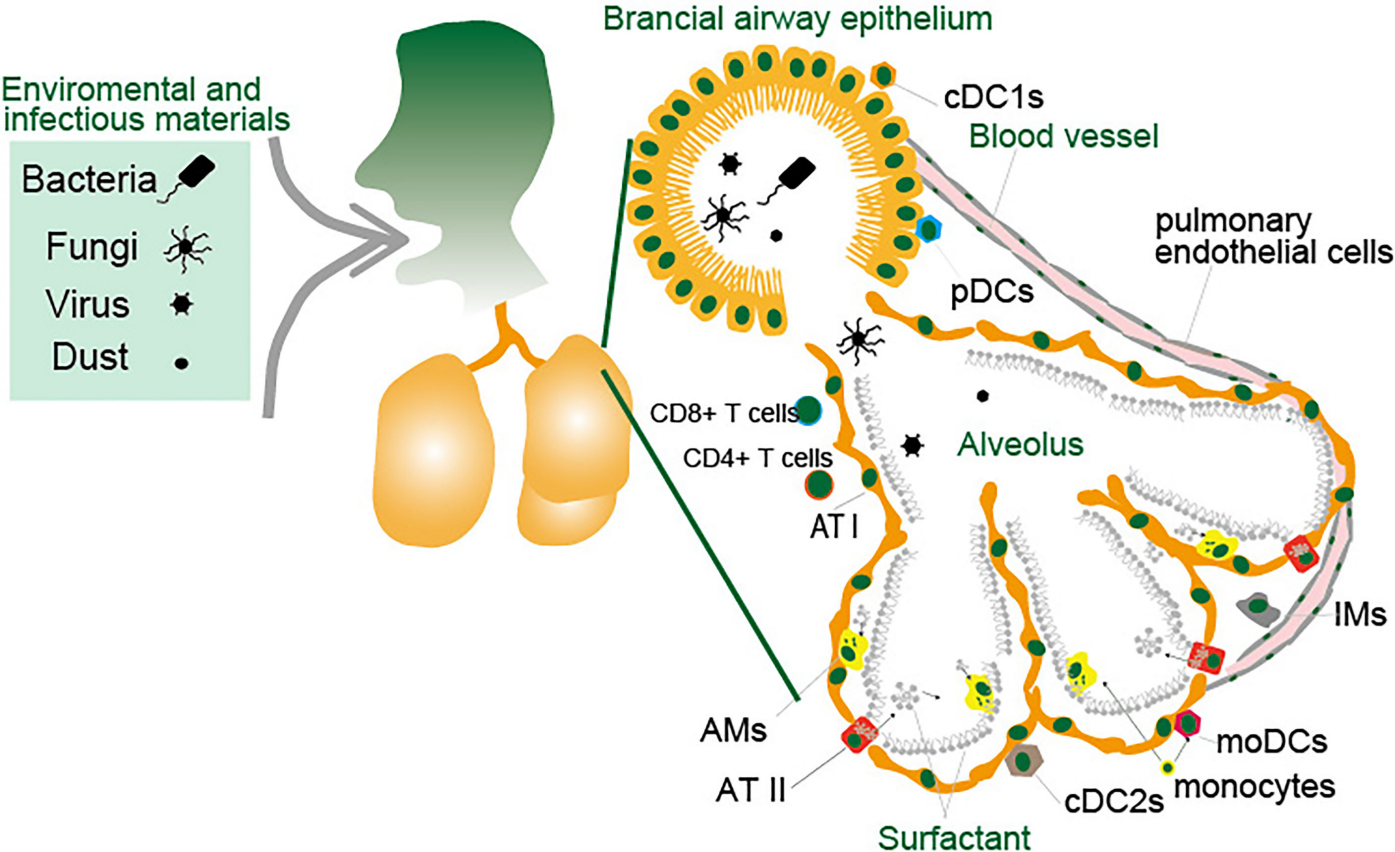
Antigen presenting cells in the lung. The lungs are constantly exposed to environmental and infectious agents such as dust, viruses, fungi, and bacteria that invade the lungs upon breathing. The lungs are protected by various types of immune cells and epithelial cells. Lung DCs are largely divided into three major subsets and are broadly subdivided into pDCs, cDC1s and cDC2s. MACs in the lungs consist of two major populations: AMs and IMs. LECs consist of ATI and ATII cells in the alveoli, and the endothelial cells and other types of cells constituting the bronchial airway epithelium. Monocytes migrate to the lungs in response to inflammatory stimuli in a CCR2-dependent manner and these cells differentiate to moDCs or AMs. Small blood vessels allow oxygen to be extracted from the air into the blood, and carbon dioxide to be released from the blood into the air. The cells lining the inner surface of blood vessels are the pulmonary endothelial cells. These cells function as APCs that engulf pathogens through endocytosis or phagocytosis and present their peptides on major MHC-I or MHC-II protein complex to CD8^+^ or CD4^+^ T cells.

The lung is composed of a complex tissue structure that exchanges gas and is exposed to outer space. The combination of crosstalk between DCs and MACs effectively protects against inhaled pathogens by inducing acquired immunity (**Figure 3**). cDC1s, cCD2s and IMs express high levels of MHC I/II with co-stimulatory molecules CD80 and CD86 (106). However, AMs express lower levels of MHC-II. Based on the expression of molecules for antigen presentation, it is revealed that each cells display antigen presentation capacity against specific infectious pathogens and allergic materials in the lungs.

#### Antigen Presentation by pDCs

pDCs are professional cells that secrete type I IFN through the stimulation of innate immune receptors. It is widely accepted that the production of type I IFN by pDCs in the lungs is important for host defense against pathogens. An *Aspergillus fumigatus* infection model in the lung demonstrated that pDCs are essential for host defense and neutrophil effector activity (107). Antigen presentation by pDCs in the lungs is controversial during pathogen infection. Resting pDCs are weak antigen-presenting cells, but appear to be functionally specialized for their ability to capture and present viral antigens to CD4^+^ T cells in the presence of CpG DNA or virus stimulation (108, 109). Transplantation of pDCs in an IAV infection model showed that pDCs infected with IAV promote antigen presentation to CD8^+^ T cells (110). In contrast, ablation of pDCs does not have a significant impact on the production of IAV-specific CD8^+^ T cells and viral clearance, indicating that pDCs have weak or no antigen cross-presentation capacity *in vivo* (111). Other groups have shown that pDCs in other peripheral tissues cooperate with cDC2s to promote their maturation and cross-presentation activity and induce antiviral CD8^+^ T cells, suggesting that pDCs indirectly induce antigen-specific CD8^+^ T cells (112, 113).

#### Antigen Presentation by cDC2s

cDC2s are localized in the lungs under a steady-state condition, and a large number of cDC2s are accumulated in the lungs in response to inflammation induced by viral infection (114) or antigen immunization (115). IAV infection induces accumulation of cDC2s, and the depletion of these cells reduces the number of virus-specific CD8^+^ cells and mortality (85–87). These results indicate that accumulated cDC2s migrate to the lymph nodes and present antigens to CD8^+^ T cells. However, cDC1 analysis using *Batf3*-deficient mice indicated that cDC2s have a weak cross-presentation capacity *in vivo* and support the proliferation of CD8^+^ T cells in the lung during IAV infection (116). Initial antigen-specific T cell differentiation is induced in the tissue-draining lymph nodes, and lung cDC2s are less migratory than cDC1s (117). During the inflammation, cDC2s in the lungs have shown to prime CD4^+^ Th2 cells but not CD8^+^ T cells responses (69, 75, 76). cDC2s also have shown to prime CD4^+^ Th17 cells response during *Aspergillus fumigatus* infection (74). T follicular helper (Tfh) cells are a subset of CD4^+^ T cells that promote antibody production during vaccination. cDC2s carry antigen into the lymph node where cDC2-dependent Tfh cells prime antibody-mediated protection from IAV challenge (67). cDC2s also locate in lymphoid organ, skin intestine and others organs as same with lung cDC2s, and cDC2s in the other organs efficiently promote the differentiation of CD4^+^ T cells into effector helper T cells during infection with *Nippostrongylus brasiliensis*, *Aspergillus fumigatus* or *Citrobacterior rodentium* (70–73). These results suggest that cDC2s are more specialized in polarizing CD4^+^ T helper cell responses and providing help to B cells, rather than in inducing CD8^+^ T cells activation.

cDC2s consist of heterogeneous subpopulations although it is unclear whether the same subpopulation of cDC2s induces both Th2 and Th17 cells (71, 74, 94). Single-cell RNA and cytometry by time-of-flight (CyTOF) analyses revealed that cDC2s consist of five distinct clusters. Ly-6C^+^CD301b^–^ cDC2s promote Th17 differentiation, and CD200^+^cDC2s induce the differentiation of Th2 but not Th17 cells (94). In addition, there are conflicting reports on how moDCs and CD11b^+^DCs interact with and regulate T cell responses (118). A recent report indicated that inflammatory cDC2s (inf-cDC2s) express the Fc receptor CD64 shared with moDCs and IRF8 shared with cDC1s and are infiltrated to present antigen to CD4^+^ and CD8^+^ T cells during respiratory virus infection (92). TNFR2^−^ cDC2 subpopulation drives moDCs maturation to generate T follicular helper (Tfh) cells in the lung (119).

#### Antigen Cross-Presentation by cDC1s

Many studies have shown the importance of cDC1s in the initiation of antiviral T cell response following influenza infection. Particular subsets of cDC1s, such as CD8α^+^ and CD103^+^ cDC1s, play specific roles in naïve T cell activation and differentiation (10, 120–122). CD8α^+^ cDC1s in the spleen and lymphoid organs are known as the cross-presenting subset (123–125). CD103^+^ cDC1s are migratory DCs that cross-present antigens in peripheral tissues, including the lungs (126, 127). Both CD103^+^ cDC1s in the lungs and CD8α^+^ cDC1s in lymph nodes share the expression of various genes, including transcription factors IRF8, BATF3, and ID2, and both of these DC subtypes are developed in the presence of Flt3 (128).

cDC1s directly present antigen to naive CD4^+^ T cells (129) and cDC1s could prime Th2 and Th17 differentiation by producing IL4, IL12, IL13 and IL17 induction during allergic airway inflammation (130, 131). A mouse model of invasive pulmonary aspergillosis infection showed cDC1s induces Th17 response by producing IL-2 in the lung (132). Other reports postulate that cDC1s promote airway tolerance by the induction of FoxP3^+^ T_reg_s in antigen induced airway inflammation (133) or by inducing IL-10 without T_reg_-induction (134). Although cDC1s can present antigens and stimulate CD4^+^ T cells, they are well known for their ability to cross-present antigens to CD8^+^ T cells (127, 132). Lung cDC1s preserve viral antigens in their endocytic compartments and control the induction of virus-specific CD8^+^ T cells through antigen cross-presentation (116, 135). Lung cDC1s migrate to mediastinal LNs after viral infection, where they directly present antigens to naïve CD8^+^ T cells or transfer captured antigens to CD8α^+^ cDC1s, which present antigens and activate naïve CD8^+^ T cells (86, 136, 137). In addition to cDC1s, cDC2s have the potential to migrate to mediastinal LNs (MLNs) (117), however, cDC2s do not present antigens efficiently in the MLNs (138). The cytotoxic activity of CD8^+^ T cells plays a critical role in viral clearance in the lungs. Initial virus-specific CD8^+^ T cells in the LNs are induced by cDC1s migrating from the infected lung, and the virus-specific CD8^+^ T cells then traffic back to the infected lung to mediate their effector function (10, 11, 139).

#### Antigen Presentation by moDCs

Chemokine receptor CCR2- and Ly6C-expressing inflammatory monocytes infiltrate into the lung during pathogen infection including *Aspergillus fumigatus* (140) and IAV (141), and differentiate rapidly into moDCs. MoDCs in other organs are also capable of presenting antigen and priming to CD4^+^ T cells (142, 143) and CD8^+^ T cells (88). However, the precise function of moDCs to regulate T cells response in lung is controversial. CCR2-deficient mice impair moDCs recruitment and exhibit reduction of effecter CD8^+^ T cell response in the lung after IAV infection (85). moDCs depletion by CD11c-cre-*Irf4* ^f/f^ mice reduces CD8^+^ memory precursor cells and T_RM_ cells during IAV infection (144). MoDCs in the lung prime IFN-γ-producing antigen-specific CD4^+^ T cells in pulmonary *aspergillosis* (140). MoDCs also promote Th1 and Th17 cell polarization through antigen presentation during allogeneic responses (118) and induce Th2 type CD4^+^ cells during house dust mite allergy (145). Report using CD26 as a maker for separation of moDCs indicated that moDCs have poor capacity to migrate to lymph node and prime CD4^+^ T cells and CD8^+^ T cells (92, 117, 146).

#### Antigen Presentation by Macrophages

AMs develop during embryogenesis, and then predominantly maintain their populations by self-renewal (147–149) and are specialized in the removal and recycling of surfactant molecules. Although AMs are the most abundant immune cells in the lungs and have been suggested to play a functional role in antigen presentation during tuberculosis and Cryptococcus neoformans infection in humans (150, 151), supportive evidence for antigen presentation by AMs has not been reported in mice. Certain IM subsets have been contributed to lung immune homeostasis by spontaneously producing the immunosuppressive cytokine IL-10 and preventing the development of aberrant type 2 allergic responses against inhaled allergens (101, 104). IMs are separated by a distinct subpopulation based on the surface expression pattern (103, 152) and single-cell RNA sequencing (153, 154), some of which express antigen-presenting genes and may mediate antigen presentation to CD4^+^ T cells in the lungs. Accumulated Ly-6C^+^ monocytes develop to exudative macrophages (exMACs) during *Cryptococcus* (155), *Streptococcus* (156) and IAV infection (157). ExMACs produce high levels of TNF-α and NOS2 and stimulate the proliferation of memory CD4^+^ T cells (157).

### Antigen Presentation by Monocytes

Two types of monocytes have been identified with different phenotypes and functions: Ly6C^+^ classical monocytes and Ly6C^−^ non-classical monocytes. Ly6C^+^ monocytes constitutively enter to lung tissues in the steady state and a large number of these cells migrate to the lungs in response to inflammatory stimuli in a CCR2-dependent manner (158, 159). Ly6C^+^ monocytes develop to moDCs, IMs, exMACs or monocyte-derived AMs in the lungs during inflammatory stimulation, but in the steady state, monocytes continuously migrate to non-lymphoid organs including lung without differentiating into other types of cells and may exit lung *via* the lymphatics or undergo local apoptosis and cleared (160). Ly6C^+^ monocytes have been shown to produce large amounts of IL-1, IL-6, and TNFα, and have an ability to drive adaptive immune responses through antigen presentation (160). Ly6C^+^ monocytes in other tissues reported that these cells have an ability to present antigen to both CD4^+^ and CD8^+^ cells. Ly6C^+^ monocytes regulate early host response to *Aspergillus* lung infection by taking up conidia and trafficking them into the draining LN to prime CD4^+^ T cells (140). Cross-presentation by Ly6C^+^ inflammatory monocytes in lymphoid organs has been reported in the presence of TLR agonists, especially TLR7 (161). Once recruited into the lungs, Ly6C^+^ monocytes further differentiate into moDCs and monocyte-derived AMs. Recent evidence have shown that CCR2-deficient mice, which are defective in monocyte trafficking to the lung, exhibit decreased number of virus-specific lung resident memory CD8^+^ (T_RM_) cells by the antigen presentation on monocytes (162).

### Antigen Presentation by Epithelial and Endothelial Cells

As lung epithelial cells directly interact with the external environment, these cells are thought to be critical regulators of barrier immunity (163, 164). The alveoli are composed of two distinct lung epithelial cell types: AT I cells, which are thin and cover approximately 95% of the internal surface of the lung, and AT II cells, which are cuboidal secreting cells located between type I cells (165). AT I cells are specialized in gas exchange and alveolar fluid regulation, whereas type II cells secrete surfactants and constitute the progenitor cells of the epithelium (166). There is increasing evidence that epithelial cells in the lung contribute to adaptive immune responses in the lungs. AT II cells express MHCII and present antigen. *In vitro* co-culture experiments AT II cells with antigen specific hybridoma suggested that AT II cells activate CD4^+^ cells to induce IFNγ in the presence of peptide antigen, and deletion of MHC-II on AT II cells results in a modest worsening of respiratory virus disease following influenza and Sendai virus infections (167). Surfactant Protein C (SPC)^low^MHC-II^high^ AT II cells function as APCs to induce CD4^+^ T_RM_ cells (7). Antigen presenting AT II cells primes naïve CD4^+^ T cells *in vitro* and induce regulatory T (T_reg_) cells (168); however, it is unclear whether AT II cells prime naïve CD4^+^ T cells *in vivo* (169). In addition to CD4^+^ T cells activation, barrier epithelial cells recruit and maintain CD8^+^ T_RM_ cells near the sites of antigen encounter and reactivate them in the tissues *via* local antigen presentation (12, 170).

Small blood vessels, known as capillaries, come in close contact with the alveoli, allowing oxygen to be extracted from the air into the blood, and carbon dioxide to be released from the blood into the air. The cells lining the inner surface of these capillaries are known as the pulmonary endothelial cells (171). Lung endothelial cells cross-present malaria antigen to antigen specific reporter cells *in vitro* and a mouse model of malaria infection by *Plasmodium berghi* ANKA (PbA) induces IFNγ positive CD8^+^ T cell. These results demonstrate that lung endothelial cells cross-present malaria antigen to CD8^+^ T cells, although it is unclear whether these cells activate naive CD8^+^ T cell *in vivo* (172).

## Perspective and Conclusion

Lungs are protected by various types of APCs that stimulate antigen-specific CD4^+^ and CD8^+^ T cells against infectious pathogens. cDC1s and cDC2s work as professional APCs in the lung. Sub-population of cDCs has been investigated by deep separation using single RNA sequence and CyTOF technology and have shown to process and present antigen. In addition, there has been increasing evidence for antigen presentation by resident APCs such as epithelial cells, epithelial cells in the lungs. The relation of pathogen and inflammation model to APCs was shown in **Table 1**. Although MACs express MHC and costimulatory molecules with higher engulfment capacity, the role of MACs in the lung as APCs is still unclear.

**Table 1:** Lung APCs and their roles in T cell responses.

Model		T cell type		Reference
Influenza virus	cDC2	Tfh		Krishnaswamy et. al. (67)
	pDC	CD8		Hemann A. et. al. (110)
	cDC1	CD8		Helft et. al. (116), Waithman et. al. (135), Low et. al. (12), Jenkins et. al. (139), Kim and Braciale (10), Kohlmeier et. al. (11)
	moDCs	CD8 TRM		Ainsua-Enrich et. al. (144)
	epithelial cells	CD4		Toulmin et. al. (167)
	epithelial cells	CD8 TRM		Wein et. al. (170), Low et. al. (12)
	monocyte	CD8 TRM		Dunbar et.al. (162)
House dust mite	cDC2	CD4 (Th2)		Tussiwand et. al. (75), Williams et. al. (76)
	cDC2	CD4 (Th2,Th17)		Tussiwand et. al. (75), Izumi et. al. (94)
	moDC	CD4 (Th2)		Plantinga et.al. (145)
Aspergillus fumigatus	monocyte	CD4		Hohl et. al. (140)
	cDC2	CD4 (Th17)		Schlitzer et. al. (74)
	cDC1	CD4 (Th17)		Zelante et. al. (132)
Mycoplasma pneumonia	cDC1	CD8		Sun et. al. (127)
Pneumonia virus	cDC2	CD8, CD4		Bosteels et. al. (92)
Maralia(Plasmodium berghi ANKA)	endothelial cell	CD8		Claser et. al. (172)
Lung inflammation by transgenic mice	epithelial cells	CD4		Gereke et. al. (168)

To initially prime antigen-specific T cells, antigen-captured DCs and migratory APCs need to traffic to lung-draining LNs where they encounter naïve T cells to select antigen-specific T cells. Following a program of proliferation and differentiation of T cells in LNs, antigen-specific effector or memory T cells migrate back to the infected lung to mediate their effector function (10, 11). At the same time, antigen-specific effector or memory T cells are reactivated by APCs, including monocytes, epithelial cells and endothelial cells in the lungs, with support of cytokine production and the local microenvironment (12). Among the antigen specific memory type cells, CD4^+^ and CD8^+^ T_RM_ cells in the lung provide protection against pathogen infection and retain for long time period in the peripheral tissue. Pulmonary antigen encounter is necessary for the establishment of T_RM_ during IAV infection in the lung (173), and antigen presentation by DCs with cytokines such as TGF-β and IL15 is shown to be important for T_RM_ development in the lung (174–176). Various types of APCs in the lungs contribute to pathogen clearance against viruses, fungi, and bacteria; therefore, APCs perform their function depending on the pathogen infection, and further studies are needed to clarify the role of individual APCs in the lungs.

## Author Contributions

TakK and MI wrote the manuscript. TarK edited and supervised the manuscript. All authors contributed to the article and approved the submitted version.

## Conflict of Interest

The authors declare that the research was conducted in the absence of any commercial or financial relationships that could be construed as a potential conflict of interest.

## Publisher’s Note

All claims expressed in this article are solely those of the authors and do not necessarily represent those of their affiliated organizations, or those of the publisher, the editors and the reviewers. Any product that may be evaluated in this article, or claim that may be made by its manufacturer, is not guaranteed or endorsed by the publisher.
